# Novel PNKP mutations associated with reduced DNA single‐strand break repair and severe microcephaly, seizures, and developmental delay

**DOI:** 10.1002/mgg3.2295

**Published:** 2023-11-02

**Authors:** Ann‐Charlotte Thuresson, Jan Brazina, Talia Akram, Julia Albrecht, Niklas Dahl, Cecilia Soussi Zander, Keith W. Caldecott

**Affiliations:** ^1^ Department of Immunology, Genetics and Pathology, Science for Life Laboratory Uppsala Uppsala University Uppsala Sweden; ^2^ Genome Damage and Stability Centre University of Sussex Brighton UK; ^3^ Department of Pediatrics Falun Hospital Falun Sweden; ^4^ Present address: Departments of Pediatrics, Neurology and Physiology Northwestern University Feinberg School of Medicine, Ann & Robert H. Lurie Children's Hospital of Chicago Chicago Illinois USA

**Keywords:** developmental delay, MCSZ, microcephaly, PNKP, seizures, single‐strand break repair, SSBR

## Abstract

**Background:**

Microcephaly with early‐onset seizures (MCSZ) is a neurodevelopmental disorder caused by pathogenic variants in the DNA strand break repair protein, polynucleotide kinase 3′‐phosphatase (PNKP).

**Methods:**

We have used whole genome sequencing and Sanger sequencing to identify disease‐causing variants, followed by a minigene assay, Western blotting, alkaline comet assay, γH2AX, and ADP‐ribose immunofluorescence.

**Results:**

Here, we describe a patient with compound heterozygous variants in *PNKP*, including a missense variant in the DNA phosphatase domain (T323M) and a novel splice acceptor site variant within the DNA kinase domain that we show leads to exon skipping. We show that primary fibroblasts derived from the patient exhibit greatly reduced levels of PNKP protein and reduced rates of DNA single‐strand break repair, confirming that the mutated *PNKP* alleles are dysfunctional.

**Conclusion:**

The data presented show that the detected compound heterozygous variants result in reduced levels of PNKP protein, which affect the repair of both oxidative and TOP1‐induced single‐strand breaks, and most likely causes MCSZ in this patient.

## Graphical abstract

Microcephaly with early‐onset seizures (MSCZ) is a neurodevelopmental disorder caused by pathogenic variants in the DNA strand break repair protein, polynucleotide kinase 3′‐phophatase (PNKP). Here, we describe a patient with compound heterozygous variants in PNKP, one missense variant, and a novel splice acceptor site variant which leads to exon skipping. We show that primary fibroblasts derived from the patient exhibit greatly reduced levels of PNKP protein and reduced rates of DNA single‐strand break repair, confirming that the mutated PNKP alleles are dysfunctional.

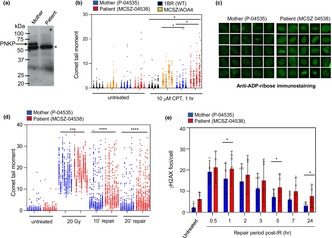

## 1 INTRODUCTION

Polynucleotide kinase 3′‐phosphatase (*PNKP*; MIM#605610) is a DNA end‐processing enzyme that restores DNA termini present at oxidative and topoisomerase 1 (TOP1)‐induced DNA breaks to canonical 3′‐hydroxyl and 5′‐phosphate moieties (Caldecott, 2022; Jilani et al., 1999; Karimi‐Busheri et al., 1999). PNKP interacts with the DNA strand break repair scaffold proteins XRCC1 and XRCC4 during DNA single‐strand break repair (SSBR) and non‐homologous recombination (NHEJ)‐mediated DNA double‐strand break repair (DSBR), respectively (Chappell et al., 2002; Koch et al., 2004; Loizou et al., 2004; Whitehouse et al., 2001). PNKP is comprised of three domains: a forkhead‐associated domain that interacts with XRCC1 and XRCC4 and facilitates PNKP recruitment to DNA breaks, a central DNA phosphatase domain that dephosphorylates 3′‐phosphate termini, and a C‐terminal DNA kinase domain that phosphorylates 5′‐hydroxyl termini (Bernstein et al., 2005; Caldecott, 2003). Mutations in *PNKP* might thus impede both SSBR and DSBR, though to date pathogenic variants associated with *PNKP*‐related disorders have been shown to greatly affect only SSBR (Kalasova et al., 2020).

Strikingly, pathogenic variants in *PNKP* have been associated with three distinct neurological diseases (Caldecott, 2022), microcephaly with early‐onset seizures and developmental delay (MCSZ; MIM#613402; Shen et al., 2010), ataxia‐oculomotor apraxia‐4 (AOA4; MIM#616267; Bras et al., 2015; Poulton et al., 2012), and Charcot–Marie–Tooth disease type 2B2 (CMT2B2; MIM#605589) (Leal et al., 2018). Here, we describe a 6‐year‐old boy with MCZS and compound heterozygous variants in *PNKP*, one of which is a novel splice‐site variant (c.1299‐3C>G) and the second of which (c.968C>T; p.Thr323Met) is a previously reported missense variant (Lindy et al., 2018; Vázquez et al., 2020). By employing primary fibroblasts derived from the patient, we show that these variants result in impaired SSBR, further strengthening the link between defects in this process and hereditary neurological disease.

## 2 MATERIALS AND METHODS

### 2.1 Ethical compliance

This study was approved by the Uppsala Ethical Review Board 2019–03596 and 2020–06592 and has been performed according to Helsinki's declaration. A written informed consent was obtained from the parents of the index patient.

### 2.2 Case report

**FIGURE 1:**
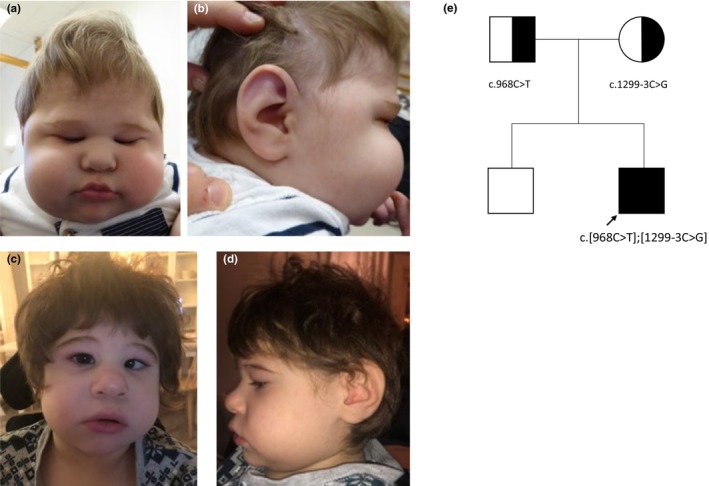
Patient at the age of (a, b) 10 months and (c, d) 2 years and 10 months. (e) Pedigree of the family.

The proband is a 6‐year‐old boy who was born at gestational age 39 + 1, as the second child of non‐consanguineous parents. There was no family history of neurodevelopmental disease. Birth weight was 2800 g (−2 SD), length 47 cm (−2 SD), and head circumference 31 cm (−3 SD). Soon after birth, the parents felt something was not right as the boy looked small and had a very small head. MRT performed at age 3.5 months showed a widened subarachnoid space with supratentorial wide sulci; reduced gyration with shallow sulci; a slight dilation of the lateral ventricles, particularly the anterior and temporal horns; a thin corpus callosum; hypoplasia of the inferior vermis and pons. No heterotopia was detected. Early development was reported as normal. At the age of 6 months, the boy presented with seizures and was diagnosed with infantile spasm at 9 months of age. EEG revealed multifocal epileptiform activity consistent with the diagnosis of hypsarrhythmia. Treatment with Synacthen (tetracosactide) and ketogenic diet showed no improvement at that time. Clinical examination at 11 months revealed a psychomotor retardation, microcephaly (−9 SD), straight eyebrows, small palpebral fissures, flat nasal bridge, high insertion of columella, hypoplastic helices, cone‐shaped fingers with prominent fingertip pads, short and overriding toe (digII) (Figure 1). Height and weight were −3 SD. At 2 years and 10 months, the boy could roll over, sit by himself, and stand on all fours. The boy cannot stand upright nor walk, but he smiles and reacts to physical stimuli such as tickling. He had tense Achilles tendons. The seizures were variable in type and frequency. Focal emotional seizures with laughing and absence seizures often appeared 3 days in a row, followed by a week of no seizures. Generalised tonic clonic seizures (GTC) appeared approximately twice per month. At 5 years of age, he has around 10 absence seizures a day. In addition, he also has seizures where he makes sounds (smacks), turned blue and vomit, with a duration of about 30 s, mainly during the night, and sometimes, he presents GTCs. Growing older, he has developed a spasticity mainly in his legs and receives Botox treatment in his hip adductors and in m. gastrocnemius. He is followed according to the national follow‐up surveillance program for people with cerebral palsy since around the age of 4.5. At age 5.5, he has a tendency of bilateral hip subluxation. The boy has a hyperopic astigmatism, esotropia of the left eye, and abnormal visual behaviour. VEP performed at 3.5 years of age shows some transmission along the visual axes. From 1 year old, he has been fed by gastric tube and has a diahorrea lasting 1 to 2 weeks every third month but has improved after change of nutrient solution. His current growth parameters are −3 SD in height and weight and a head circumference of −7 SD. His growth parameters seem to level out and his seizures tend to get worse when he is eating more. He has a severe intellectual disability, hyperactivity, shows no signs of dystonia, but presents some dyskinetic movements. He exhibits repetitive movements like clapping his hands, hitting his head and enjoys jumping when sitting in a swing. He is non‐ambulatory and can no longer sit by himself, nor stand on all fours, but enjoys rolling around on the floor. Recently, he had a hip surgery due to his spasticity. He cannot talk but is reacting on a limited number of signs and sounds and is fond of music. He is not interested in pictures/screens and does not reach for or grasp at objects. A chromosomal microarray analysis performed at 5 months of age showed normal results.

### 2.3 Genetic analysis

The patient and parents were recruited to a large‐scale sequencing study of individuals with intellectual disability and epilepsy. Genomic DNA was extracted from peripheral blood leucocytes according to standard procedures. Trio WGS was performed using Illumina's PCR‐free protocol (TruSeq DNA Sample prep kit–Illumina), HiSeqX system (Illumina Inc.), and v2 sequencing chemistry to at least 30x coverage using standard protocols. Sequence reads were mapped to human reference genome Hg19 as previously described (Halvardson et al., 2016; Zhao et al., 2018). Variant filtering and prioritisation of variants were performed using Alissa Interpret (Agilent technologies) to identify disease‐causing variants in the proband. Identified variants were verified by Sanger sequencing using standard protocols, and subsequently, classified according to the American College of Medical Genetics and Genomics (ACMG) guidelines (Richards et al., 2015). Patient data have been submitted to DECIPHER. (https://decipher.sanger.ac.uk/).

### 2.4 In vitro splicing assay

Minigenes containing the wild‐type (WT) and mutated PNKP allele (MT) were constructed from PCR amplicons of genomic DNA from the proband spanning exon 14 to 16 of *PNKP* as previously described (Akram et al., 2020). In brief, amplicons were cloned into a pCR4‐TOPO vector (Invitrogen) and constructs containing the PNKP amplicons were confirmed by sequencing. Inserts from each allele were then excised and cloned into the pAcGFP1‐N1 expression vector (Clontech) and subsequently transfected separately into HeLa cells. Total RNA extracted from the transfected HeLa cells were then analysed by RT‐PCR and gel separated to compare the size of the RT‐PCR products followed by cDNA sequencing. Primer sequences are available on request.

### 2.5 Cell lines and culture

The unaffected parent (mother) and affected proband (son) primary fibroblasts were obtained with consent from a skin biopsy and are denoted P‐04535 (unaffected mother) and MCSZ‐04538 (affected son). The unrelated 1BR primary human fibroblasts and patient‐derived AOA4/MCSZ fibroblasts have been described before (Hoch et al., 2017; Kalasova et al., 2020). The fibroblasts were grown in DMEM/F12 medium (MERCK) supplemented with antibiotics and 20% fetal bovine serum (MERCK) and maintained in a low‐oxygen incubator (3%).

### 2.6 Alkaline comet assays (SSB repair assays)

DNA strand breaks were measured by alkaline comet assay (Breslin et al., 2006). In brief, primary fibroblasts were trypsinised, washed, and resuspended in growth medium (1–1.5 x 10^5^ cells per sample) and then either treated with DMSO vehicle or 10 μM camptothecin (MERCK) for 45 min at 37°C, or cooled on ice for 5 min and exposed to γ‐rays (20 Gy, on ice) and incubated in a water bath at 37°C to allow DNA repair. Samples were then placed on ice at the indicated timepoints and processed as described below. Cell suspensions were centrifuged, washed, and resuspended in 450 μL ice‐cold PBS, and 150 μL of the cell suspension mixed with 150 μL low‐melting agarose (1.2% in PBS at 42°C). A total of 150 μL of the mixture was spread on a microscopic glass slide, lysed for 60 min in cold (4°C) alkaline lysis buffer (2.5 M NaCl, 100 mM EDTA, 10 mM Tris pH 10, 1% DMSO (added immediately before use) and 1% Triton X‐100), and then washed and equilibrated for 45 min in cold (4°C) alkaline electrophoresis buffer (1 mM EDTA, 50 mM NaOH, ph 13). Cells were then subjected to electrophoresis for 25 min at 12 V. Slides were then neutralised (400 mM Tris‐Cl pH 7.4) for 30 min and stained in a solution containing SYBR Green (1/10,000; MERCK) and antifade (45 μg/mL) for 15 min. Samples were scored blind (Comet Assay IV software, Perceptive Instruments).

### 2.7 γH2AX and ADP‐ribose immunofluorescence

Primary fibroblasts were seeded at 40%–50% on coverslips in petri dishes (TPP 93040) and when 80%–90% confluent were γ‐irradiated (2 Gy) at room temperature and incubated (37°C) for the indicated times to allow repair. Coverslips were then rinsed in PBS and fixed in 4% formaldehyde/PBS (20 min, RT), rinsed in PBS, and incubated in pre‐extraction buffer (25 mM Hepes pH 7.4, 50 mM NaCl, 0.5% Tx‐100, 300 mM sucrose, 1 mM EDTA, 3 mM MgCl_2_) for 5 min at room temp, and then rinsed in PBS and blocked in 5% BSA (in PBS) for 60 min at RT. Coverslips were stained in anti‐γH2AX (1:2500 in 5%BSA/PBS) primary antibody for 60 min at RT, washed 3x in PBS, and stained with a goat anti‐mouse secondary antibody (1:1000 in 5% BSA/PBS, Invitrogen A21235). Coverslips were washed 3x in PBS, incubated in DAPI/water solution (5 min, RT), washed in water, dried (lightproof box, RT), and mounted using Vectashield (H‐1000, Vector laboratories). Automated wide‐field microscopy was performed on a scanR high‐content imaging platform (Olympus) with scanR Image Acquisition and Analysis Software using 40 x/0.95NA dry objective (UPLSAPO 2 40×). For anti‐ADP‐ribose immunofluorescence, fibroblasts seeded on coverslips and grown as above, but in 24‐well dishes, were incubated with 10 μM camptothecin (MERCK) for 45 min in the presence/absence of 10 μM PARG inhibitor (PDD00017273, MERCK). Coverslips were then washed in ice‐cold PBS and incubated with ice‐cold pre‐extraction buffer (2 min on ice), washed in cold PBS, and fixed in 4% formaldehyde/PBS (20 min, RT) and processed as described above in γH2AX staining section. Macro Green reagent was used for PAN staining (final concentration 1 μg/mL).

### 2.8 Western blotting

Fibroblasts for Western blotting were lysed in 2x sample buffer (125 mM Tris pH 6.8, 4% SDS, 20% glycerol) and denatured at 90°C for 3 min. Protein concentration was measured using a BCA kit (ThermoFisher). DTT (50 mM final concentration) and bromophenol blue were added to samples prior to further heating (3 min at 90°C) and SDS‐PAGE. Proteins were transferred to nitrocellulose membrane (ThermoFisher) in Towbin buffer, blocked (5% milk in TBST), and incubated with the anti‐PNKP primary antibody (GeneTex 129,606; 1:1000) followed by HRP‐conjugated secondary goat anti‐rabbit antibody (DAKO, P0448).

## 3 RESULTS

### 3.1 Molecular genetics

**FIGURE 2:**
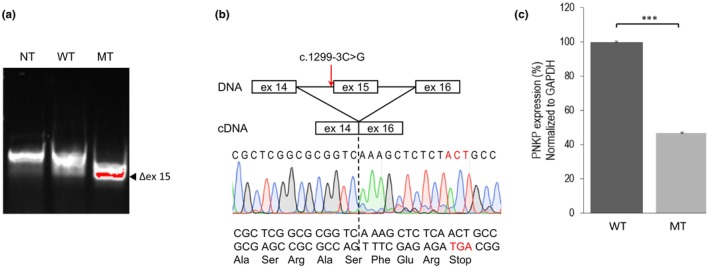
In vitro splicing assay. (a) Gel‐separated RT‐PCR products of minigene constructs encoding genomic DNA spanning exon 14–16 from WT PNKP and the splice‐site variant c.1299‐3C>G (MT), showing a reduced product size for the MT compared to WT. (b) Sanger sequencing of cDNA derived from the above mRNA products of the WT and MT minigenes, confirming skipping of exon 15 of *PNKP*. Skipping of exon 15 is predicted to result in a frameshift and a premature stop codon (highlighted in red). (c) qRT‐PCR of steady state mRNA levels expressed from the WT and MT.

Trio WGS identified compound heterozygous variants in the *PNKP* gene (NM_007254.4) in the proband. The variants were identified as the missense variant c.968C>T [p.Thr323Met], located in exon 11, and the putative splice acceptor site variant c.1299‐3C>G, located in intron 14. The variant c.968C>T [p.Thr323Met] was paternally inherited and has been reported previously in MCSZ (Lindy et al., 2018; Vázquez et al., 2020). Thr323 is located in the DNA phosphatase domain of PNKP and is highly conserved. Thr323Met has an MAF of 4.54 × 10^−5^ (gnomAD v3.1) and is predicted to be deleterious by SIFT, probably damaging by Polyphen2, disease causing by MutationTaster, and has a CADD score of 24.6. Indeed, Thr323Met may result in an unstable PNKP protein (Jiang et al., 2022), similar to what has been reported for several other disease‐associated PNKP‐mutated alleles (Kalasova et al., 2019, 2020; Reynolds et al., 2012). In contrast, the maternally inherited variant c.1299‐3C>G is a predicted acceptor splice‐site variant (SpliceAI: ΔS acceptor gain = 0.85, ΔS acceptor loss = 0.47). c.1299‐3C>G is a novel variant that has not been reported previously and is located within the DNA kinase domain of PNKP. It is not present in gnomAD v3.1 and has a CADD score of 23. To analyse the impact of the novel predicted acceptor splice‐site variant c.1299‐3C>G on PNKP splicing, a minigene assay (Cooper, 2005) was performed in which genomic DNA spanning exons 14–16 of the WT and variant PNKP (MT) alleles was amplified, subcloned into an expression vector, and transfected into HeLa cells. Following expression and PCR amplification, the minigene cDNA products of the WT and MT PNKP alleles were separated by gel electrophoresis. The WT cDNA amplicon was of the size predicted for properly spliced exons 14–16, whereas the cDNA amplicon from the MT allele was shorter and of a size that could be consistent with exon 15 skipping (Figure 2a). Indeed, sequence analysis of the gel‐separated cDNA amplicons confirmed exon 15 skipping in transcripts from the mutated *PNKP* allele (Figure 2b). Hence, skipping of exon 15 will most likely result in a frameshift and a premature stop codon at the fifth amino acid downstream of the breakpoint. Furthermore, quantification by qRT‐PCR of mRNA levels expressed from the WT and c.1299‐3C>G minigene alleles revealed that mRNA levels derived from the mutated allele were markedly reduced (Figure 2c).

**FIGURE 3:**
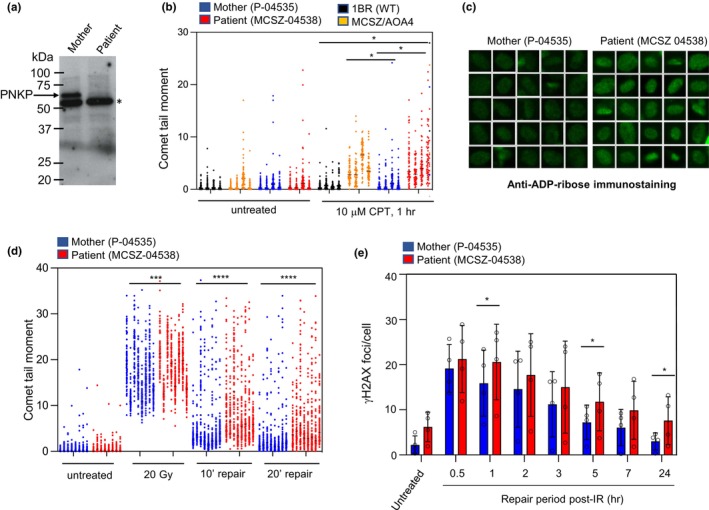
Reduced rates of SSBR in primary fibroblasts from a proband with MCSZ. (a) Western blot analysis showing reduced PNKP protein levels in primary fibroblasts derived from the current patient (MCSZ‐04538) and his unaffected mother (P‐04535). Asterisk denotes a non‐specific band detected by the antibody. (b) Levels of CPT‐induced DNA strand breaks measured by alkaline comet assays in the indicated primary fibroblasts prior to (“untreated”) and following treatment for 1 h with 10 μM CPT. Data are the individual comet tail moments (an arbitrary measure of DNA strand breakage) of 100 cells per sample for *n* = 4 experiments, with each experiment plotted side by side. Significantly different pair‐wise comparisons are shown, as determined by two‐ANOVA with Sidak's multiple comparison test with matching applied for samples within experimental repeats (*p < 0.05). (c) ADP‐ribose levels measured by immunofluorescence in parental (mother) and patient primary fibroblasts following treatment with CPT. Representative single‐cell galleries are shown, from scanR high‐content imaging. (d) Levels of γ‐ray‐induced DNA strand breaks measured by alkaline comet assays in wild type (mother, P‐04535) and patient (son, MCSZ‐04538) primary fibroblasts prior to irradiation (“untreated”), immediately after irradiation (20 Gy), and following incubation for the indicated times (10–20 min) to allow repair. Data are the individual comet tail moments of 100 cells per sample for *n* = 8 independent experiments, with each experiment plotted side by side. The datasets for mother and patient were significantly different (*p* < 0.0001) as determined by two‐ANOVA with Sidak's multiple comparison test, with matching applied for samples within experimental repeats. Significantly different pair‐wise comparisons are shown (ns, not significant, ***p < 0.001; ****p < 0.0001). (e) Levels of DSBs measured by γ‐H2AX immunostaining before and at the indicated times after γ‐irradiation (2 Gy). The indicated wild type (1BR, 04535) and patient (AOA4/MCSZ, 04538) primary fibroblasts were γ‐irradiated (2Gy) and incubated for the indicated times to allow DSB repair. Data are the mean γH2AX foci per cell (an indirect measure of the number of DSBs per cell), from *n* = 4 independent experiments. The data sets for mother and patient were significantly different (*p* < 0.0001) as determined by two‐ANOVA with Sidak's multiple comparison test, with matching applied for samples within experimental repeats. Significantly different pair‐wise comparisons are shown (*p < 0.05).

Previous analyses revealed that PNKP mutations that cause MCSZ often greatly affect the level of PNKP protein in primary patient fibroblasts, either by reducing levels of properly spliced mRNA as above and/or by destabilising the mutant protein (Kalasova et al., 2019, 2020; Reynolds et al., 2012). To see if this was true for the PNKP variants identified in the current patient, we isolated primary fibroblasts and conducted Western blotting. Consistent with other MCSZ patients, primary fibroblasts derived from the proband (denoted MCSZ‐04538) exhibited greatly reduced levels of PNKP protein, when compared to the unaffected mother (denoted P‐04535) (Figure 3a).

Next, we examined whether the PNKP mutations described here affected the rate of DNA strand break repair in patient cells. We reported previously that cells from MCSZ patients typically exhibit reduced rate of repair of SSBs induced as a result of the abortive activity of topoisomerase 1 (TOP1): a physiologically relevant source of SSBs that are implicated in neurological disease and are substrates for both the DNA kinase and DNA phosphatase activities of PNKP (Kalasova et al., 2019, 2020; Reynolds et al., 2012). To examine if this was the case for the current patient, we treated the parental P‐04535 and MCSZ‐04538 primary fibroblasts with the TOP1 poison camptothecin (CPT) and measured the accumulation of DNA strand breaks using alkaline comet assays. Whilst this assay can detect both SSBs and DSBs, we have shown previously that it is not sufficiently sensitive to detect CPT‐induced DSBs that accumulate as a result of defects in NHEJ: the DSBR pathway in which PNKP is involved (El‐Khamisy et al., 2005). Consequently, following CPT treatment, this assay detects only the role of PNKP in SSBR. As expected, we saw little increase in the steady‐state level of CPT‐induced SSBs in an unrelated normal fibroblast cell line (1BR) and in fibroblasts from the unaffected mother (P‐04535), reflecting the SSBR proficiency of these cells (Figure 3b). In contrast, we detected a reproducible accumulation of CPT‐induced SSBs in MCSZ‐04538 fibroblasts from the proband and in fibroblasts derived previously from a patient with combined MCSZ/AOA4 (Figure 3b). We also detected elevated levels of poly (ADP‐ribose) in MCSZ‐04538 cells following CPT treatment (Figure 3c), when compared to parental P‐04535 cells, which reflects increased activity of the SSB sensor protein PARP1 and is another indicator of reduced rates of SSBR in PNKP‐mutated patient cells (Hoch et al., 2017; Kalasova et al., 2019, 2020).

We next employed alkaline comet assays to compare the ability of parental and patient fibroblasts to rapidly repair DNA strand breaks induced by oxidative stress, many of which are substrates for the DNA 3′‐phosphatase activity of PNKP. Treatment with γ‐rays (20‐Gy) resulted in high levels of DNA breakage in all of the primary fibroblast cell lines employed (Figure 3d). However, although the DNA breaks declined nearly to background levels within 20 min following irradiation in the parental P‐04535 fibroblasts, those in the MCSZ‐04538 fibroblasts declined at a much slower rate (Figure 3d). Since >95% of ionising radiation‐induced DNA breaks are SSBs (Bradley & Kohn, 1979), these data are indicative of a defect in the repair of oxidative SSBs (Figure 3d).

Finally, to measure specifically the repair of oxidative DSBs, which are substrates for the DSB repair pathway in which PNKP operates, we quantified the induction and loss of γH2AX: a robust and reliable marker of DSBs following ionising radiation (Löbrich et al., 2010). Whilst we did detect a small overall reduction in the rate of repair of DSBs induced by ionising radiation, this reduction was significant only at some time points (Figure 3e).

## 4 DISCUSSION

In this study, we report a patient with microcephaly, seizures, and intellectual disability and with compound heterozygous variants in the DNA repair gene, *PNKP*. The *PNKP* variants are c.968C>T, p.(Thr323Met), and c.1299‐3C>G and are located within the DNA phosphatase and DNA kinase domains of PNKP, respectively. c.1299‐3C>G is a novel variant that has not previously been reported, and we show here that this variant induces skipping of exon 15, leading to deletion and translational frameshift within the DNA phosphatase domain and most likely to nonsense‐mediated RNA decay. Consistent with this finding, a homozygous mutation of this same splice acceptor site at an adjacent nucleotide (c.1299‐2A>G) similarly causes skipping of exon 15 and MCSZ (Taniguchi‐Ikeda et al., 2018). Importantly, we detected little or no PNKP protein in the proband fibroblasts, in line with observations in other MCSZ cell lines. In contrast to the splice‐site variant, the missense variant T323M likely reduces PNKP levels by impacting on protein stability, as previously reported for other MCSZ‐associated missense variants, including the closely located E326K variant (Jiang et al., 2022; Reynolds et al., 2012; Shen et al., 2010).

The variant c.968C>T, p.(Thr323Met) has previously been reported in a homozygous state in an 11 month‐old male patient (Vázquez et al., 2020). Apart from microcephaly, seizures, and developmental delay, both present with brain abnormalities, are non‐ambulatory, and cannot talk (Table 1), Neither this and our patient presented with ocolumotor apraxia, which was reported for the homozygous variant c.1299‐3C>G (Taniguchi‐Ikeda et al., 2018). However, the oculomotor apraxia presented first in the teenage age and might not be present yet in our patient.

**TABLE 1:** Summary of clinical findings in patients with the variants c.968C>T and c.1299‐3C>G in *PNKP*.

	Our patient	Taniguchi‐Ikeda et al	Vázquez et al.
*PNKP* variant (NM_007254.4)	c.[968C>T];[1299‐3C>G]	c.[1229‐2A>G];[1229‐2A>G]	c.[968C>T];[968C>T]
Gender	Male	Female	Male
Age	6 y	38 y	11 m
Developmental delay	Severe	Severe	+
Seizures	+	+	+
Brain MRI	Widened subarachnoid space with supratentorial wide sulci, reduced gyration with shallow sulci, slight dilation of the lateral ventricles, thin corpus callosum, hypoplasia of the inferior vermis and pons	Spinocerebellar degeneration	Cerebral atrophy with thin corpus callosum and reduced cerebral white matter volume, simplified gyral pattern, cerebellar hypoplasi
Absent speech	+	ND	+
Inability to walk	+	ND	+
Microcephaly	−7 SD	−7.8 SD	−10 SD
Oculomotor apraxia	−	+ (as teenager)	−
Short stature	−3 SD	−3.8 SD	+
Facial dysmorphology	Straight eyebrows, small palpebral fissures, flat nasal bridge, high insertion of columella, hypoplastic helices	ND	Depressed nasal bridge, sloping forehead, short neck
Other	Cone‐shaped fingers with prominent fingertip pads, short and overriding toe	Oesophageal atresia, clubfeet, hypoalbuminemia, frostbite	Hypotonia

*Note*: + = present, − = absent, m = months, ND = not determined, SD = standard deviation, y = year.

Primary fibroblasts from the proband exhibited reduced rates of repair of SSBs arising from either oxidative stress or abortive TOP1 activity, both of which are physiologically relevant sources of DNA breaks and are substrates for PNKP (Caldecott, 2022). This is consistent with our previous work, in which cells from other MCSZ patients similarly exhibit reduced repair of both oxidative and TOP1‐induced SSBs, resulting primarily from their greatly reduced levels of PNKP protein (Kalasova et al., 2019, 2020; Reynolds et al., 2012). However, it is most likely that it is unrepaired oxidative SSBs that cause the neurodevelopmental seizures and microcephaly that typify MCSZ, rather than TOP1‐induced SSBs. This is because cells from patients with AOA4 and CMT2B2, which lack these severe neurodevelopmental pathologies, appear to be proficient for repair of oxidative SSBs (Kalasova et al., 2020). AOA4 and CMT2B2 cells do however exhibit reduced rates of repair of TOP1‐induced SSBs, which we have shown primarily reflects the loss of DNA 5′‐kinase activity, arguing that it is the latter that causes progressive neurodegeneration in these diseases. A prediction of this model is that MCSZ patients in whom the repair of both oxidative SSBs and TOP1‐induced SSBs is defective should exhibit not only neurodevelopmental pathology resulting from unrepaired oxidative SSBs but also neurodegeneration and/or cerebellar ataxia resulting from unrepaired TOP1‐induced SSBs that typifies AOA4 and CMT2B2. Whilst cerebellar ataxia was not initially reported in initial MCSZ patients, it has recently been reported in some cases (Entezam et al., 2018; Gatti et al., 2019; Poulton et al., 2012; Rudenskaya et al., 2019; Taniguchi‐Ikeda et al., 2018). It will thus be important to assess the current and other MCSZ patients in future, for cerebellar dysfunction and/or other neurodegenerative pathology.

Finally, we also detected a small reduction in the rate of repair of DSBs induced by ionising radiation, though this was not significant at all time points. The lack of a robust defect in DSB repair is consistent with our previous data, but is surprising because PNKP is involved in DSB repair (Chappell et al., 2002; Kalasova et al., 2020) mediated by non‐homologous end‐joining (NHEJ) and cells with defects in NHEJ typically exhibit much slower rates of DSB repair (Kalasova et al., 2020). Perhaps the small defect detected here reflects the presence of residual PNKP protein in the patient cells. This would be consistent with the small increase in DSB repair defect observed in MCSZ cells following depletion of the residual PNKP using siRNA, though this effect was again very small and not statistically significant (Kalasova et al., 2020). Alternative explanations are that the role of PNKP in DSB repair is partially or largely redundant with that of another 3′‐end‐processing protein, as has been suggested previously (Chalasani et al., 2018), or that the small increase in detected DSBs reflects the defect in SSBR, which can result in the conversion of SSBs to DSBs during DNA replication.

## AUTHOR CONTRIBUTIONS

Contributors Ann‐Charlotte Thuresson, Jan Brazina, and Keith W. Caldecott designed and planned the study. Ann‐Charlotte Thuresson, Jan Brazina, Talia Akram, Niklas Dahl, and Keith W. Caldecott analysed the data. Jan Brazina and Talia Akram performed experimental lab work. Julia Albrecht, Cecilia Soussi Zander, and Ann‐Charlotte Thuresson diagnosed the patients, performed clinical assessments, and collected patient samples. Ann‐Charlotte Thuresson and Keith W. Caldecott wrote the manuscript. All authors read, commented on, and approved the manuscript.

## FUNDING INFORMATION

ACT and CSZ were supported by Uppsala‐Örebro Regional Research Council (RFR‐649321) and ALF‐grants from Uppsala University Hospital, Sweden. JB and KWC were supported by an MRC Programme Grant to KWC (MR/W024128/1).

## CONFLICT OF INTEREST STATEMENT

The authors declare no conflict of interests.

## ETHICS APPROVAL STATEMENT

This study was approved by the Uppsala Ethical Review Board 2019‐03596 and 2020‐06592 and has been performed according to Helsinki's declaration.

## PATENT CONSENT STATEMENT

A written informed consent was obtained from the parents of the index patient.

## Data Availability

Patient data has been submitted to DECIPHER (#405651). Data analysed during this study are included in this published article. Raw data is available upon request.
